# Genomic and Immunological Characterization of Pyroptosis in Lung Adenocarcinoma

**DOI:** 10.1155/2022/6905588

**Published:** 2022-07-27

**Authors:** Yaobo Song, Zhen Qu, Hu Feng, Long Xu, Yajie Xiao, Zhikun Zhao, Dongfang Wu, Chao Sun, Xinglong Fan, Dongmei Zhou

**Affiliations:** ^1^Department of Medical Oncology Ward, Yantaishan Hospital, Yantai 264001, China; ^2^Department of Oncology, No. 970 Hospital, Yantai, 264001, China; ^3^Department of Oncology, Weihai Municipal Hospital, Weihai, Shandong 264200, China; ^4^Department of Oncology, General Hospital of Northern Theater Command, Shenyang 210100, China; ^5^Department of Clinical Translational Medicine, YuceBio Technology Co., Ltd., Shenzhen 518000, China; ^6^Department of Thoracic Surgery, Qilu Hospital (Qingdao), Cheeloo College of Medicine, Shandong University, Qingdao 266035, China

## Abstract

Pyroptosis is a programmed cell death that may either promote or hinder cancer growth under different circumstances. Pyroptosis-related genes (PRGs) could be a useful target for cancer therapy, and are uncommon in lung adenocarcinoma (LUAD). The expression profiles, mutation data and clinical information of LUAD patients were included in this study. A pyroptosis-related prognostic risk score (PPRS) model was constructed by performing Cox regression, weighted gene co-expression network analysis (WGCNA), and least absolute shrinkage and selection operator (LASSO) analysis to score LUAD patients. Somatic mutation and copy number variation (CNV), tumor immunity, and sensitivity to immunotherapy/chemotherapy were compared between different PPRS groups. Clinical parameters of LUAD were combined with PPRS to construct a decision tree and nomogram. Red module was highly positively correlated with pyroptosis. Seven genes (FCRLB, COTL1, GNG10, CASP4, DOK1, CCR2, and AQP8) were screened from the red module to construct a PPRS model. Significantly lower overall survival (OS), higher incidence of somatic mutation and CNV, elevated infiltration level of the immune cell together with increased probability of immune escape were observed in LUAD patients with higher PPRS, and were more sensitive to Cisplatin, Docetaxel, and Vinorelbine. We constructed a new PPRS model for patients with LUAD. The model might have clinical significance in the prediction of the prognosis of patients with LUAD and in the efficacy of chemotherapy and immunotherapy.

## 1. Introduction

Pyroptosis is a type of cell death programmed caused by the family of proteins known as Gasdermin, which results in cell enlargement, dissolution of plasma membranes, fragmentation of chromosomes, and release of intracellular pro-inflammatory molecules, thereby triggering inflammation and immune responses [1–3]. The relationship between cancer and pyroptosis is a prominent subject in immunology at present. Pyroptosis has a crucial role in enhancing or inhibiting several cancers types, including breast cancer, gastric cancer, esophageal cancer, cervical cancer [4]. In addition, cancer cell pyroptosis can be induced during cancer therapy, including chemotherapy, the treatment by small molecule drugs, and nanodrugs [5]. Recent studies focused on those affecting pyroptotic inflammasomes and promoting pyroptosis molecules, which are expected to be effective targets for the treatment of different cancers [4].

In non-small cell lung cancer (NSCLC) cell lines, simvastatin was found to suppress cancer cell proliferation and migration through inducing pyroptosis [6]. Especially, the gasdermin D (GSDMD) and gasdermin E (GSDME) are two important executioners in the pyroptosis mechanism induced by cancer therapy [7]. A number of pyroptosis core proteins are associated with prognosis of many cancer types such as hepatocellular carcinoma, colorectal cancer, gastric cancer, and lung cancer [3]. Gao et al. discovered that knocking down GSDMD could restrict NSCLC cell growth both in vitro and in vivo, and GSDMD overexpression was significantly associated with poor prognosis in lung adenocarcinoma (LUAD) [8].

To date, several bioinformatics-based studies have identified pyroptosis-related genes (PRGs) in specific cancers. Chen et al. developed a risk model consisting of 6 PRGs, which can successfully be used to evaluate the survival and prognosis of hepatocellular carcinoma and distinguish the risk and predict the immune infiltration and treatment efficiency of HCC [9]. Recent reports provide a novel PRGs signature to predict breast cancer patients' tumor immune microenvironment and prognosis [10]. Zhou's study identified a group of PRGs that can effectively predict ovarian cancer patients' response to chemotherapy and immunotherapy [11]. Luo et al. screened seven possible biomarkers to predict the prognosis of patients with colorectal cancer and provide therapy recommendations for these patients [12]. Lung cancer is a leading cause of cancer-related deaths [13], with lung adenocarcinoma (LUAD) accounting for 40% of the incidence of all lung cancer cases [14]. The role of PRGs depends on the type of cancer, and few PRGs have been found in LUAD. Therefore, PRGs play an important role in LUAD.

By performing PRGs bioinformatics analysis, we investigated the LUAD genetic variation on the basis of PRGs in the present research. The pyroptosis-related prognostic risk score (PPRS) model was developed based on the least absolute shrinkage and selection operator (LASSO) and Weighted gene co-expression analysis (WGCNA) regression analysis. The features of differential mutation, biological process, immune infiltration, immunotherapy, and chemotherapy response between PPRS groups were studied. In addition, PPRS was combined with clinicopathological features in the construction of a decision tree and nomogram to optimize the predictive accuracy of the risk of LUAD.

## 2. Materials and Methods

### 2.1. Data Collection and Processing

The workflow of this study was shown in Figure S1. We obtained the expression profile of gene data, copy number variation (CNV), and somatic mutation data of the treated primary LUAD samples from The Cancer Genome Atlas (TCGA) database (https://portal.gdc.cancer.gov/). After preprocessing, 500 primary LUAD samples remained in the TCGA cohort. Two other independent LUAD cohorts, GSE31210 and GSE72094, were obtained from the Gene Expression Omnibus (GEO, http://www.ncbi.nlm.nih.gov/geo/). After preprocessing, 226 samples and 398 samples remained in the GSE31210 and GSE72094 cohorts.

### 2.2. Acquisition and Genomic Mutation Analysis of PRGs

We obtained 27 PRGs from Molecular Signature Database (MSigDB) [15] (https://www.gsea-msigdb.org/gsea/msigdb/) by searching for “REACTOME PYROPTOSIS.” The somatic mutation in LUAD tissue was shown by waterfall map by R software “maftools” [16]. Comparison of CNV difference of 27 PRGs was examined by Kruskal-Wallis test.

### 2.3. The Relation between Pyroptosis Score and LUAD Prognosis

Based on the expression level of 27 PRGs, the pyroptosis score of each sample in LUAD was quantified by ssGSEA and arranged in ascending order. The relation of pyroptosis score, clinical features (including T stage, age, N stage, M stage, sex, survival status, and clinical stage) with overall survival was evaluated by multivariate and univariate Cox regression analysis. Pyroptosis score in different clinical features was analyzed by Wilcoxon test or Kruskal-Wallis test.

### 2.4. WGCNA

With the R package “WGCNA,” a gene co-expression network was developed [17] using gene expression value in the identification of the co-expression gene module. First, the scale-free topology fit index for 1 to 30 powers was computed using the “pickSoftThreshold” function. According to blockwiseModules, automatic block module detection was performed. When the independence degree reached 0.8, the appropriate power value was determined, and the module's minimum number of genes was set to 30. Subsequently, highly related modules were merged to form a novel module (parameters: deepSplit = 2, minModuleSize = 30, height = 0.25). The highly related modules were merged into a new module (parameters: height = 0.25, deepSplit = 2, minModuleSize = 30). The correlation between eigengene module and PRGs was used to estimate the module-pyroptosis association to identify pyroptosis-related gene modules.

### 2.5. PPRS Was Constructed to Assess the Different Risks of LUAD

The hub gene of the module was identified by the gene expression of the pyroptosis-related module together with the Pearson correlation analysis on the pyroptosis score. The hub genes not substantially correlated with the survival of patients with LUAD (*P* > 0.05) were removed by performing univariate Cox regression analysis. The genes showing close correlation with the prognosis of LUAD patients were selected in performing LASSO and multivariate Cox regression analysis. Finally, the screened genes were utilized as variables in constructing the model: pyroptosis-related prognostic risk score (PPRS)=∑Coefficient(mRNAi)^*∗*^Expression i^ ^. Notably, *i* refers to the final screened genes.

### 2.6. Functional Enrichment Analysis

According to the risk score of the median, the samples were separated into two groups, namely the low-risk group and the high-risk group. The R software package “GSVA” was utilized to compute each sample's ssGSEA score in various functions, and the Pearson correlation with PPRS was analyzed for the LUAD samples in TCGA cohort. Furthermore, gene set enrichment analysis (GSEA) was performed for two groups based on candidate gene sets in the Hallmark database [15].

### 2.7. Determination of Immune Score and Stromal Score

The ESTIMATE algorithm [18] was run to estimate immune score, stromal score, and ESTIMATE score in the TME, where ESTIMATE score was the combined score of immune score and stromal score.

### 2.8. Evaluation of Immune Cell Infiltration

CIBERSORT is a gene expression-based universal deconvolution algorithm that can conduct an estimation of the relative proportion of 22 types of immune cells from tissue gene expression profiles [19]. CIBERSORT (https://cibersort.stanford.edu/) algorithm was used to assess the relative abundance of 22 types of immune cells in 3 LUAD cohorts. Moreover, the differences in the infiltration levels of immune cells between low-risk and high-risk groups were evaluated by performing the Wilcoxon rank-sum test.

### 2.9. Response Analysis regarding Immunotherapy and Chemotherapy

Based on the data of immune checkpoints obtained from HisgAtlas database [20], the differences between low-risk and high-risk groups in their expression levels got analyzed by performing the Wilcoxon test. The potential clinical effects of immunotherapy in our defined risk group were evaluated by TIDE (http://tide.dfci.harvard.edu/). Additionally, on the basis of the information retrieved from the Genomics of Drug Sensitivity in Cancer (GDSC) database, sample sensitivity to four commonly used clinical chemotherapeutic drugs was predicted. The IC50 determined by R packet “pRRophetic” was used as the comparison index among risk groups.

### 2.10. Establishment of the Decision Tree and Nomogram

According to the clinical characteristics of LUAD samples in the TCGA cohort such as age, sex, M Stage, N Stage, T stage, Stage and PPRS, decision tree was developed using the R packet “rpart”. By Combining these clinical features and PPRS, a nomogram was constructed. The model's performance was then evaluated based on the DCA using the ggDCA package.

### 2.11. Statistical Analysis

The significant differences in OS between the two PPRS groups were observed by performing the Kaplan-Meier survival analysis and log-rank test. The “timeROC” software was used to generate ROC. Wilcoxon test was used for the comparison between the two groups. All drawings and statistical analyses were carried out using the R software (R Foundation for Statistical Computing, v.4.0.0). Double-tailed *P* < 0.05 was considered to be of statistical significance.

## 3. Results

### 3.1. PRGs Expression and Genetic Differences in LUAD

**Figure 1:**
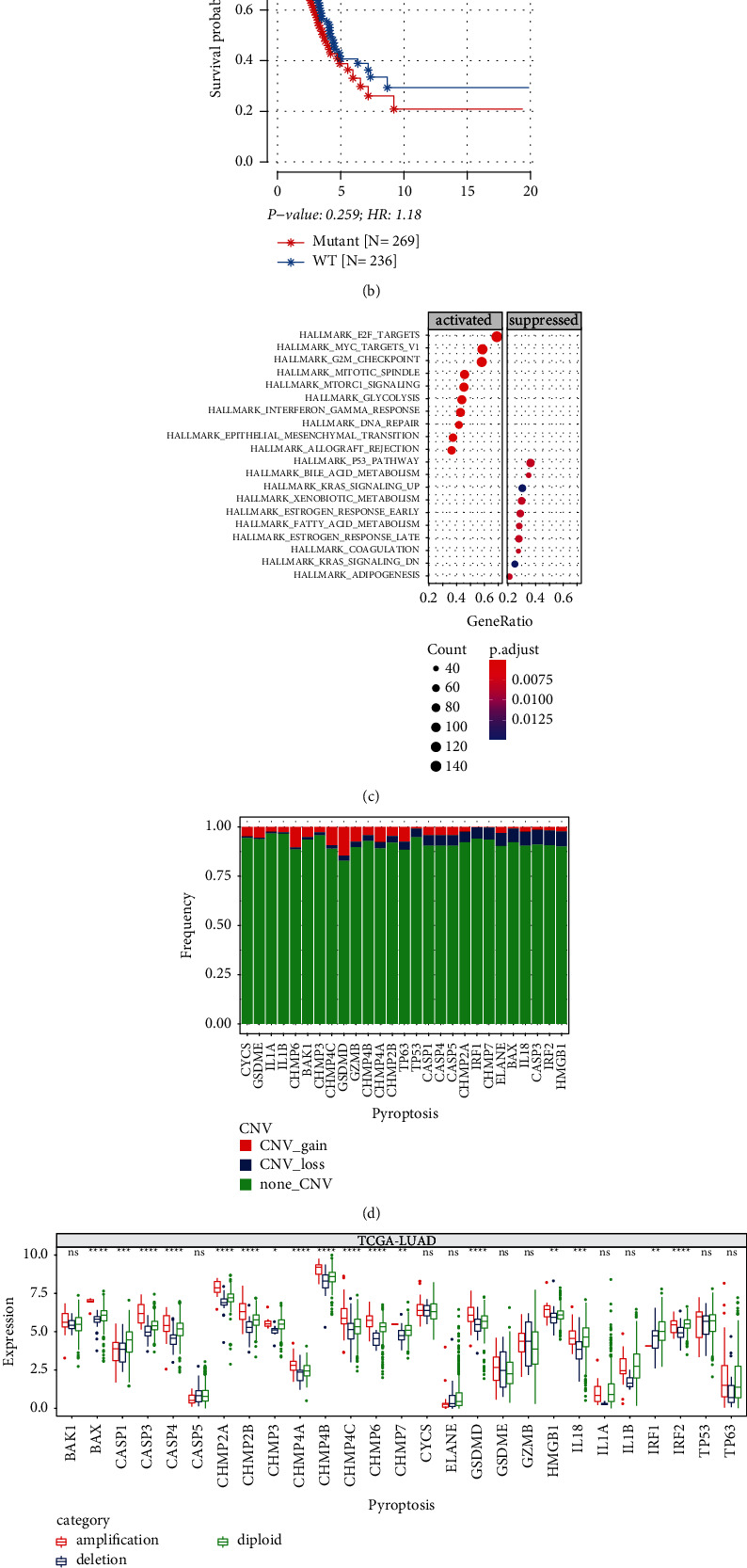
Genetic and expression variations of PRGs in TCGA cohort. (a) The waterfall diagram shows the somatic mutations of 27 PRGs from the LUAD sample of TCGA. (b) Kaplan-Meier survival plot of two groups with mutant and wild type (WT) PRGs. (c) GSEA of hallmark pathways by comparing mutant group to WT group in LUAD samples. (d) The CNV fraction of PRGs in LUAD samples. (e) Comparison of CNV difference in 27 PRGs in LUAD samples. (f) Comparison of expression of 27 PRGs between normal and LUAD samples. Log-rank test was performed in (b). Kruskal-Wallis test was performed in (e) and Wilcoxon test was performed in (f). ns, no significance. ^*∗*^*P* < 0.05, ^*∗∗*^*P* < 0.01, ^*∗∗∗*^*P* < 0.001, ^*∗∗∗∗*^*P* < 0.0001.

We investigated the presence of somatic mutations in PRGs from The Cancer Genome Atlas (TCGA, on the web: https://portal.gdc.cancer.gov/)-LUAD samples. Of the 565 LUAD samples from TCGA, 300 PRGs were mutated. The results in the waterfall showed that TP53 had the highest mutation rate (90%), followed by CASP5 (4%), TP63 (4%), CASP1 (3%), and GSDME (3%) (Figure 1(a)). To determine whether these mutations affected the survival and biological function of LUAD, the OS of PRGs wild type and PRGs mutant samples were compared, and we observed no significant change of OS between them (Figure 1(b)). The results of GSEA analysis showed that compared with PRGs wild type samples, E2F targets, MYC targets, G2M checkpoint, mitotic spindle, mTOR signaling, and DNA repair were significantly activated, whereas the p53 pathway was significantly inhibited in PRGs mutant samples (Figure 1(c)). CNV was detected in 27 PRGs, and CNV occurred in all of them, among which the copy number amplification in GSDMD, CHMP6, CHMP4C, CHMP4A, and TP6 was the most obvious, and only copy number loss occurred in CHMP2A and IRF1 (Figure 1(d)). To study whether the change of copy number had an effect on the expression of 27 PRGs, we analyzed the expression of PRGs in copy number amplification group, copy number deletion group, and copy number no significant change group, and found remarkable differences in the expression levels of these 17 PRGs among three groups (Figure 1(e)). In the 27 PRGs, apart from CASP4, CHMP6, CHMP7, GSDMD, and TP63, the other 22 PRGs were expressed differentially at a significant level between primary tumor and paracancerous samples (Figure 1(f)).

### 3.2. Pyroptosis Is the Main Factor Threatening the Survival of Lung Adenocarcinoma

We ranked the pyroptosis scores of each sample obtained by single sample gene set enrichment analysis (ssGSEA), analyzed the correlation with different clinical features, and found that pyroptosis scores were significantly correlated with N stage and survival state, and that the proportion of dead LUAD samples increased with the increase of pyroptosis scores (Figure S2A). We performed the multivariate and univariate Cox analyses to explore the influence of each pyroptosis score and clinicopathological characteristic on the prognosis of patients with LUAD. According to the results, it was observed that the prognosis of patients with LUAD was affected by the T stage, pyroptosis score, and N stage in an independent manner (Figure S2B and S2C). Moreover, the OS duration of the samples with low pyroptosis scores determined based on pyroptosis score grouping was remarkably longer compared to samples experiencing high pyroptosis scores (Figure S2D). TCGA-LUAD samples were stratified in accordance with several clinical features including T stage, N stage, survival status, clinical stage, M stage, sex, and age. From the results, it was found that the scores of pyroptosis were different at a significant level among the samples exposed to different survival conditions (either dead or alive), different N stage (N1–N4) and different clinical stage (stage I–IV) stratification, respectively (Figure S2E).

### 3.3. Identification of the Modules Associated with Pyroptosis

**Figure 2:**
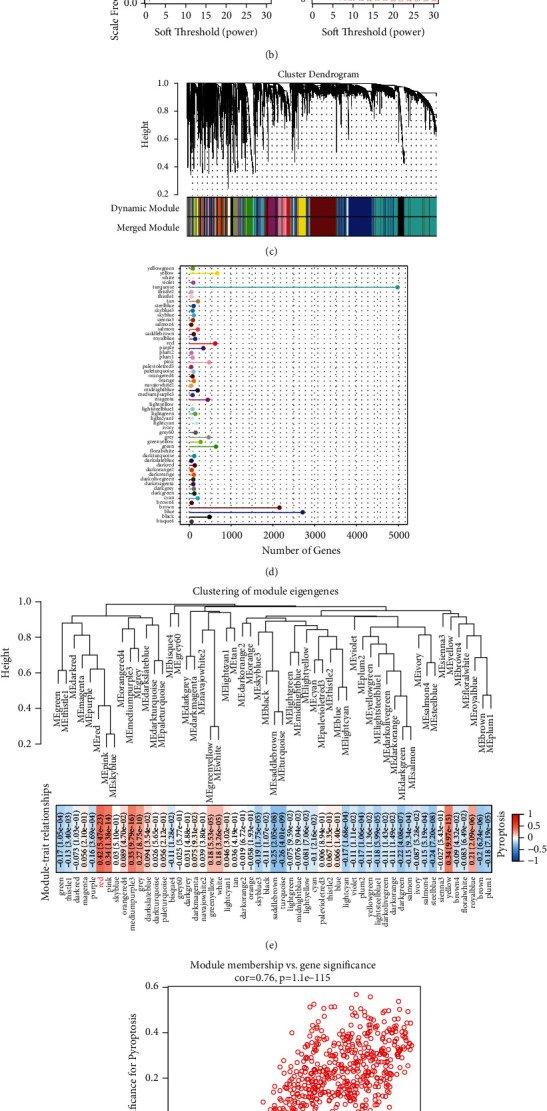
Identification of modules related to pyroptosis by WGCNA. (a) Hierarchical clustering of the genes of LUAD samples in the clustering analysis. (b) Analysis of network topology for various soft-thresholding powers. (c) The dendritic map of gene clusters generated by the dynamic tree cut package. (d) The number of genes contained in each module. (e) Correlation analysis of module-pyroptosis. The upper part represents the hierarchical clustering of the whole module, and the lower part represents the correlation between the module and pyroptosis. (f) The scatter plot of module membership (MM) and gene significance (GS) for pyroptosis in the red module.

We firstly clustered LUAD samples in the TCGA cohort to detect outliers (Figure 2(a)). The independence degree reached 0.85 and the soft threshold power was equal to 4, indicating a strong average connectivity (Figure 2(b)). The dynamic tree cut package generated a tree map of gene clusters and showed 55 modules, each of which was colored differently. (Figure 2(c)).  Figure 2(d) presented the number of genes belonging to each module. Module-pyroptosis correlation analysis displayed that there was a positive correlation between red module with pyroptosis (*r* = 0.42, *P* < 1*e* − 5) (Figure 2(e)). The module membership (MM) showed a positive correlation with gene significance (GS) for genes pyroptosis in this module (Figure 2(f)).

### 3.4. PPRS Model Construction and Evaluation

**Figure 3:**
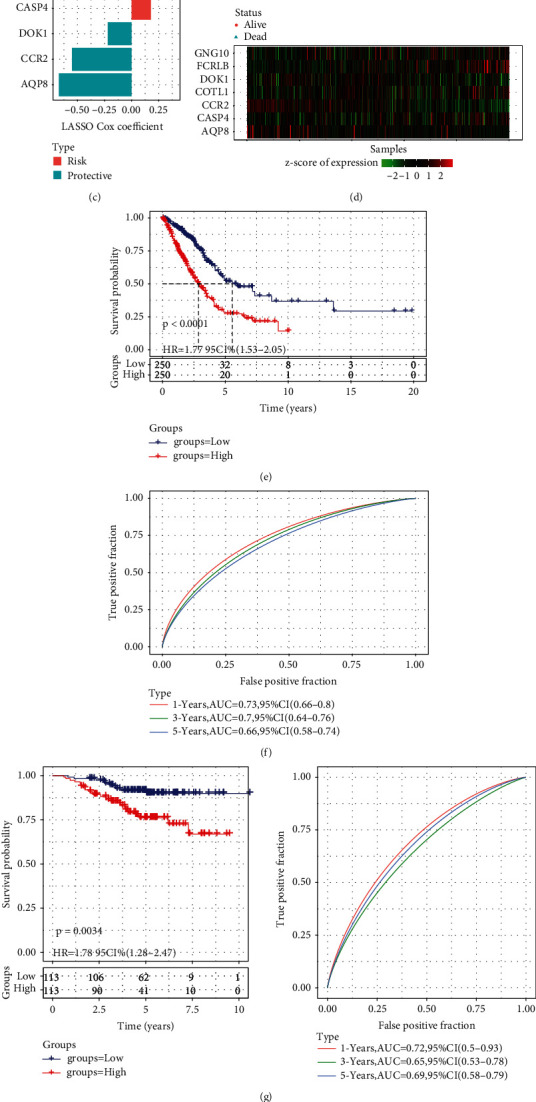
Construction and evaluation of PPRS model. (a) A total of 73 promising candidates were identified among hub genes extracted from the red module. (b) 10 of the 73 genes were retained by application of LASSO-Cox regression model with a minimum of *λ* (*λ* = 0.0295). (c) FCRLB, COTL1, GNG10 and CASP4 were risk factors, while DOK1, CCR2 and AQP8 were protective factors. (d) PPRSs and corresponding living state of the samples obtained in ascending order and expression of 7 genes of the samples. (e) The survival rate of the high-PPRS group and the low-PPRS group in the TCGA-LUAD cohort. (f) The ROC curve of the PPRS model in the TCGA-LUAD cohort. (g) In cohorts GSE31210, the difference in OS and predictive efficacy was validated. (h): The difference in OS and predictive efficacy was validated for GSE31210 cohort. Log-rank test was conducted in (e g, and h).

The genes in the red module were filtered. Specifically, the relationship between pyroptosis score and the genes in to the red module was investigated, and the module's hub genes were screened with a criterion of *P* < 0.01. A total of 73 genes were chosen for subsequent investigation using the univariate Cox regression analysis (*P* < 0.05, Figure 3(a) and Table S1). Functional analysis on these screened 73 genes showed that immune-related terms and pathways were significantly enriched, supporting a correlation between pyroptosis and tumor immunity (Figure S3). Among the 73 chosen genes, 10 of them retained with a minimum *λ* of 0.0295) by the LASSO-Cox regression model (Figure 3(b)). 7 of the 10 genes were selected to construct the model by stepwise multivariate regression analysis. Among the 7 genes, COTL1, FCRLB, CASP4, and GNG10 were the risk factors, whereas protective factors were CCR2, AQP8 and DOK1 (Figure 3(c)). The PPRS of each LUAD sample in TCGA was calculated and normalized according to our defined formula (PPRS was converted to z-score). LUAD samples were divided into high-PPRS and low-PPRS groups based on the z-score of PPRS = 0. The PPRSs of the samples were arranged in ascending order. The corresponding survival status and the expression of 7 genes showed that the increase of PPRS was accompanied by the increase in the number of dead patients (Figure 3(d)). The high-PPRS group in the TCGA-LUAD cohort had a lower survival rate at the same period, according to the results of the survival analysis (Figure 3(e)). The areas under the receiver operator characteristic (ROC) curve (AUC) for PPRS were 0.73, 0.7, and 0.66 for the 1-, 3-, and 5-year prognosis prediction, respectively (Figure 3(f)). The same analysis was performed in GSE31210 and GSE72094 cohorts, and the difference of OS between two groups and the efficacy of prediction were validated similarly, thereby confirming the overall accuracy and validity of the PPRS (Figure 3(g), 3(h)).

### 3.5. Genomic Mutation in PPRS Risk Group

**Figure 4:**
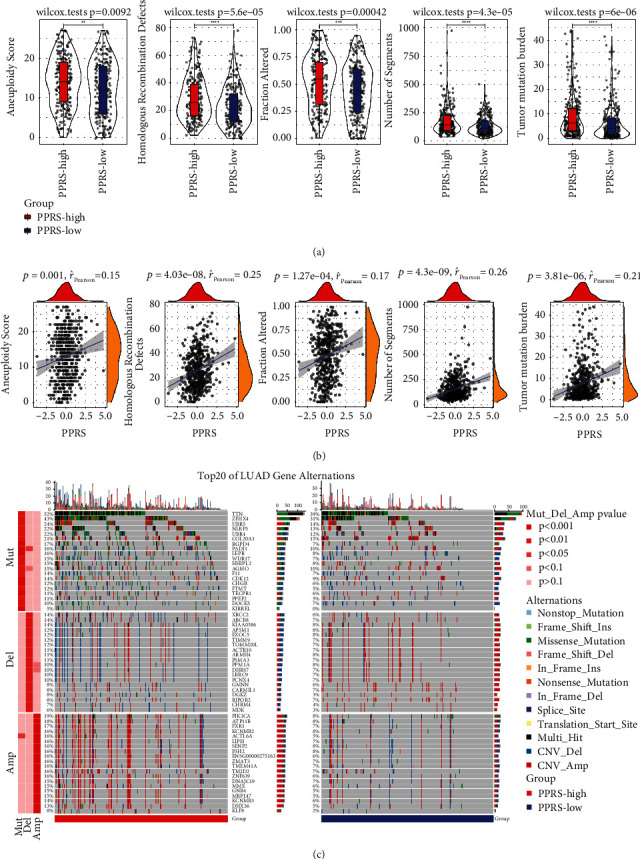
Genomic mutation in PPRS risk group. (a) Aneuploidy score, homologous recombination defects, fraction altered, number of segments and tumor mutation burden of high-PPRS group and low-PPRS group were compared by Wilcox test. (b) The relation between PPRS and aneuploidy score, homologous recombination defects, fraction altered, number of segments, tumor mutation burden, respectively. (c) The waterfall map shows the incidence of somatic mutation and copy number variation in the high-PPRS group and the low-PPRS group. ^*∗∗*^*P* < 0.01, ^*∗∗∗*^*P* < 0.001, ^*∗∗∗∗*^*P* < 0.0001.

We studied whether PPRS was related to genomic stability, and discovered that some clinical characteristics of patients belonging to the high-PPRS group were more in comparison to those in the low-PPRS group, indicating that the genome of the high-PPRS group was more unstable. This was performed through a comparison of the homologous recombination defects, the number of segments, altered fraction, the score of aneuploidy and tumor mutation burden from both groups (Figure 4(a)). All the five genomic features were positively correlated with PPRS (Figure 4(b)). The prevalence of CNV together with the somatic mutation in the high- and low-PPRS group was shown in the waterfall map. The prevalence of somatic cell mutation, CNV amplification, and deletion in the high-PPRS group was considerably higher than that in the low-PPRS group (Figure 4(c)).

### 3.6. Enrichment Pathway and Immune Characteristics of PPRS Risk Group

**Figure 5:**
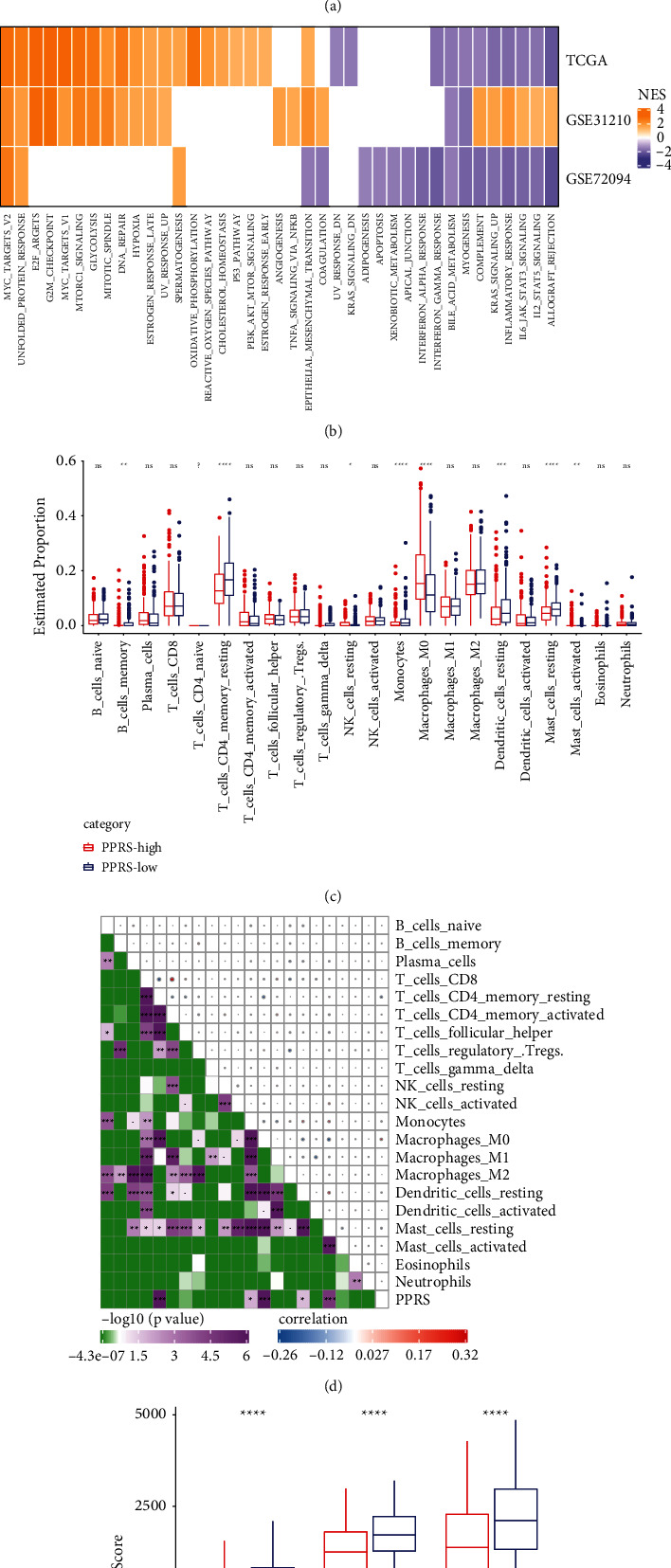
Enrichment pathway and immune characteristics of PPRS risk group. (a) Correlation analysis matrix between KEGG pathway and PPRS. (b) The NESs of high-PPRS group was higher than that of low-PPRS group. (c) The relative proportion of immune cells in TCGA-LUAD between high-PPRS group and low-PPRS group. (d) The array of Pearson correlation analysis showed the correlation between PPRS in TCGA-LUAD and 22 kinds of immune cells. (e) Stromal score, immune score and ESTIMATE score of high-PPRS group and low-PPRS group in TCGA-LUAD cohort. Wilcoxon test was conducted. ns, no significance. ^*∗*^*P* < 0.05, ^*∗∗*^*P* < 0.01, ^*∗∗∗*^*P* < 0.001, ^*∗∗∗∗*^*P* < 0.0001. ? indicates undetected expression levels.

We determined the function of PPRS and observed that there was a positive correlation between PPRS and cell cycle, replication of DNA, repair of nucleotide excision, homologous recombination, and mismatch repair and other pathways regulating cell proliferation (Figure 5(a), Table S2). We performed a comparison on the normalized enrichment scores (NESs) present in high-PPRS and low-PPRS groups, and high-PPRS was significantly enriched in MYC targets, E2F targets, G2M checkpoint, mTOR signaling, DNA repair, and other pathways relative to low-PPRS. These pathways were important pathways that affect cancer cell proliferation (Figure 5(b)). A total of 22 immune cells types were compared between the high-PPRS group and the low-PPRS group in TCGA-LUAD. We noted that the differences were quite significant in the relative proportion of resting mast cells, monocytes, M0 macrophages, resting dendritic cells, resting memory CD4T cells, resting NK cell, memory B cell as well as activated mast cells between the two groups. The relative proportion of the M0 macrophages and activated mast cells from the high-PPRS group were considerably greater than the low-PPRS group. Moreover, the relative proportion of the other 6 immune cells from the low-PPRS group was found to be higher compared to that from the low-PPRS group (Figure 5(c)). The Pearson correlation analysis array showed that PPRS was significantly correlated with resting memory CD4T cells, resting dendritic cells, M0 macrophages, monocytes, and resting mast cells (Figure 5(d)). Furthermore, immune score, stromal score, and ESTIMATE score were substantially lower in the high-PPRS group in comparison to those in the low-PPRS group (Figure 5(e)). In addition, the same tumor microenvironment (TME) analysis was also carried out in GSE31210 and GSE72094, and the results can be found in Figure S4.

### 3.7. PPRS can Assist in Identifying Patients Who Could Benefit from Chemotherapy and Immunotherapy

**Figure 6:**
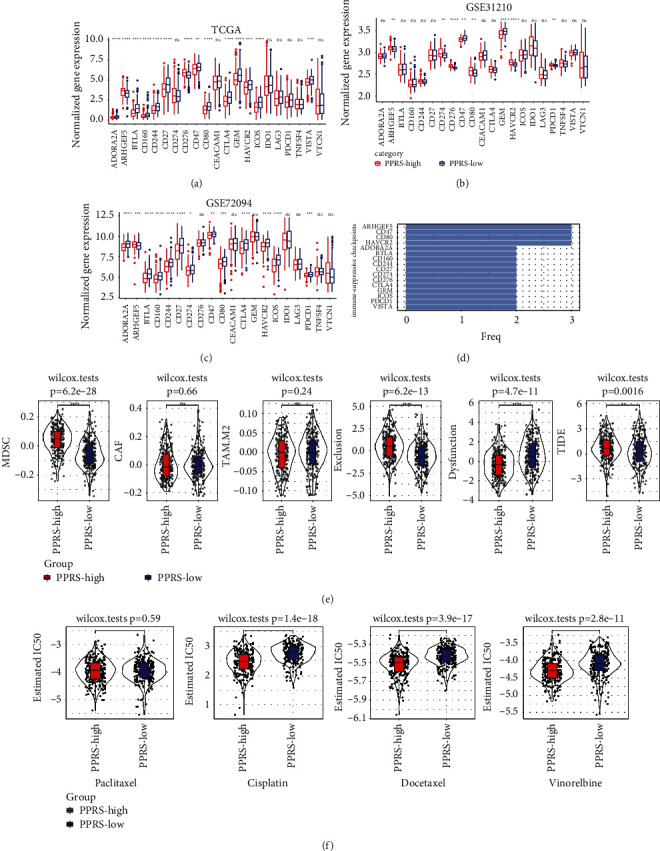
Prediction of response to immunotherapy and chemotherapy in patients with different PPRS. (a-c) The expression of 21 immune checkpoints between high-PPRS group and low-PPRS group in TCGA-LUAD (a), GSE31210 (b) and GSE72094 (c) cohorts. (d) The relative frequency of immune checkpoints expressed highest in the high-PPRS group. (e) Differences for samples with different PPRS in myeloid-derived suppressor cells (MDSC), cancer associated fibroblasts (CAF), M2 macrophages (TAM.M2), T cell exclusion, T cell dysfunction, TIDE scores. (f) Comparison for sensitivity of Paclitaxel, Cisplatin, Docetaxel and Vinorelbine in high- and low-PPRS groups of LUAD samples. Wilcoxon test was conducted. ns, no significance. ^*∗*^*P* < 0.05, ^*∗∗*^*P* < 0.01, ^*∗∗∗*^*P* < 0.001, ^*∗∗∗∗*^*P* < 0.0001.

To explore whether PPRS can distinguish the response of patients with different risks to immunotherapy and chemotherapeutic drugs, immune checkpoint expression between the two groups classified according to PPRS was analyzed. The findings indicated that many differentially expressed immune checkpoints existed in the PPRS groups of the three LUAD cohorts, and the most representative ones were CD274, CTLA4, and PDCD1, at least between the high- and the low-PPRS group of the two cohorts (Figure 6(a)–6(c)). The levels of immune checkpoints with the highest expression in the high-PPRS group were calculated by Figure 6(d). CD274, CTLA4, and PDCD1 were in the column. The scores of myeloid-derived suppressor cells (MDSC), T cell exclusion and Tumor Immune Dysfunction and Exclusion (TIDE) in the high-PPRS group were significantly higher than those in the low-PPRS group. The score of T cell dysfunction was considerably greater in the group of low-PPRS (Figure 6(e)). These findings indicated that the immune escape probability was higher in the group with high PPRS, and the potential value of immunotherapy in this group may be lower.

The half-maximal inhibitory concentration (IC50) analysis of different chemotherapeutic drugs in the group of high-PPRS and the group of low-PPRS demonstrated that the high-risk group had a lower IC50 of Docetaxel, Cisplatin, indicating that the high-PPRS group was more suitable for the treatment of Cisplatin, Docetaxel, and Vinorelbine than the low-PPRS group (Figure 6(f)).

### 3.8. The Combination of PPRS and Clinicopathological Features Improved the Survival Prediction of LUAD

**Figure 7:**
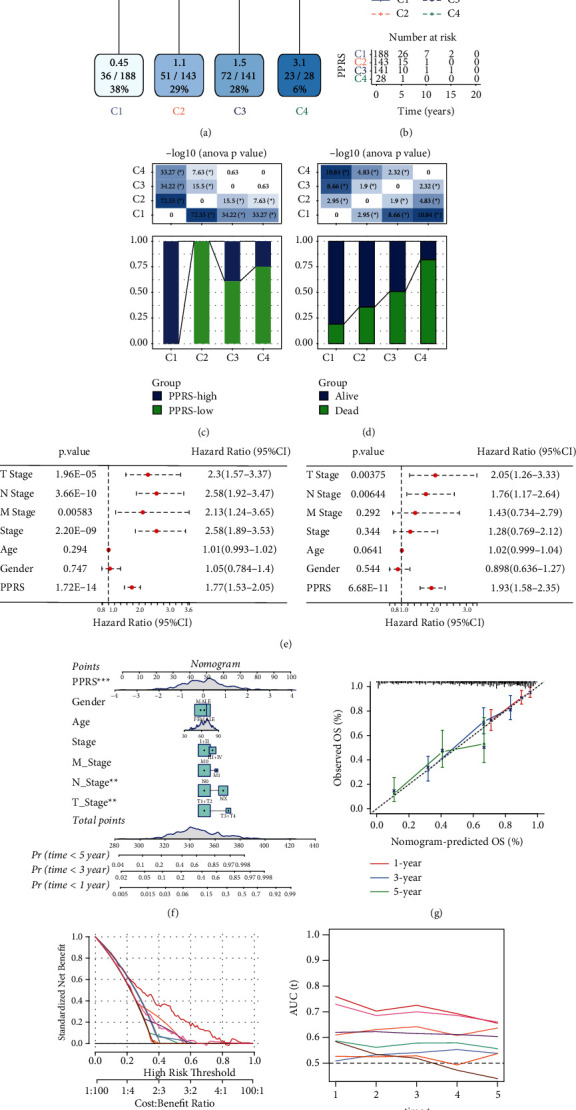
Establishment and evaluation of the decision tree and nomogram. (a) Decision trees were constructed based on age, gender, T stage, N stage, M stage, clinical stage and PPRS. (b) The survival curve of the four subgroups defined by the decision tree. (c) The distribution of PPRS in the four subgroups defined by the decision tree. (d) The proportion of patient survival and death in the four subgroups defined by the decision tree. (e) Univariate and multivariate Cox regression analysis of clinical characteristics and PPRS. (f) Nomogram constructed by combining clinical features with PPRS. (g) The calibration curve showed the consistency between the nomogram predicted OS and the actual OS in the TCGA cohort. (h) DCA of the PPRS and clinicopathological features. (i) ROC curve for clinicopathological features and nomogram. ANOVA was conducted in (c and d). Log-rank test was conducted in (e). ^*∗*^*P* < 0.05.

The decision tree on the basis of clinical characteristic (M stage, sex, age, N stage Clinical stage, and T stage) together with PPRS showed that only T stage, PPRS, and N stage were retained in the decision tree, and that four different risk subgroups C1–C4 were identified (Figure 7(a)). From C1 to C4, the risk increased gradually, the patients' OS reduced gradually, and a remarkable difference in OS between the groups was observed (Figure 7(b)). Among the four subgroups defined by the decision tree, C1 contained only low-PPRS samples, C2 only included high-PPRS samples, and C3 and C4 samples with high-PPRS accounted for a large proportion (Figure 7(c)). From C1 to C4, the proportion of patients in death status gradually increased (Figure 7(d)). Multivariate and univariate Cox regression analyses of all the PPRS and clinical features indicated that the T stage, PPRS, and N stage were the independent prognostic factors of LUAD (Figure 7(e)).

Moreover, a nomogram was constructed by combining clinical parameters with PPRS (Figure 7(f)). The calibration chart showed that the predicted OS of the nomogram fitted well with the actual OS (Figure 7(g)). Decision curve analysis (DCA) showed that nomogram was a better prognostic indicator than other variables in clinical decision-making (Figure 7(h)). The tROC analysis showed that nomogram consistently had the highest AUC in predicting 1–5 years of OS, indicating its strong ability to predict survival (Figure 7(i)).

## 4. Discussion

The unclear function of pyroptosis in cancer seems to be contextual and is dependent on genetics, cell type, and the pyroptosis induction duration [21]. The complex effects regarding pyroptosis on the onset and progression of cancer mainly included cancer cell viability inhibition, the influence of cancer cell invasion as well as migration, anti-tumor immunity enhancement, and chemosensitivity enhancement [22]. From the present research, we utilized ssGSEA and univariate and multivariate Cox regression models to identify pyroptosis as the primary risk factor for the overall survival (OS) of patients with LUAD.

Abnormal expression of some important PRGs is often observed in various types of cancer. However, most studies have focused on one or two kinds of PRG, whereas the characterization of the anti-tumor effects is usually a result of the interaction of multiple genes in a highly coordinated manner [11]. In this study, to better quantify the effect of pyroptosis on LUAD, we screened pyroptosis-related red modules by WGCNA and performed LASSO, univariate Cox regression, and stepwise multivariate Cox regression analysis for the purpose of incorporating seven genes identified in the red module for constructing a PPRS model, which can distinguish the genomic mutation and immune characteristics of patients with different PPRS, and the status of biological pathways. Several genes in the PPRS model have been reported in cancer research. Coactosin-like protein 1 (COTL1) was reported to be high-expressed in glioma tissues in the study of Shao et al. [23] and is closely correlated with the patient recurrence and prognosis. Functionally, COTL1 enhances the proliferation of cells *in vitro* and cancer growth *in vivo* [23]. A study on the peripheral blood mononuclear cells based on peripheral blood RNA-Seq indicated the GNG10 imbalance in the head and neck squamous cell carcinoma, which is related to the survival rate of patients [24]. Wang et al. detected that overexpression of GNG10 promotes the progression of colorectal cancer [25]. CASP4 expresses caspase-4 is a classical regulatory component of pyroptosis [26]. Secretoglobin 3A2-lipopolysaccharide (LPS) can eliminate human colorectal cancer cells by regulating the mechanism of CASP4-related pyroptosis [27]. Shibamoto et al. found that CASP4 expression loss is correlated with the unfavorable prognosis of patients with esophageal squamous cell carcinoma [28]. CCR2 is CC chemokine receptor 2, and CCR2 signal transduction in cancer cells can coordinate the suppression of immune response [29]. AQPs belong to a small membrane transport proteins family, whose abnormal expression plays a role in the onset and progression of several tumors [30], such as in gastric cancer [31], cervical cancer [32], and colorectal cancer [33]. These studies showed that these genes were tumor markers, and that the coordination between them was likely to have an impact on the development of LUAD.

It is reported that pyroptotic can release tumor antigens and damage-associated molecular patterns, thereby initiating adaptive immunity to enhance the efficiency of immunotherapy [34]. Herein, our analysis results showed that PPRS was not only significantly related to the classical CD274, CTLA4, and PDCD1. And MDSC, T cell exclusion, and TIDE also had significant differences in scores in different PPRS groups. Pyroptotic is also related to chemotherapy [35]. Recently published studies have shown that Cisplatin induces scorch death through activating the MEG3/NLRP3/caspase-1/GSDMD pathway in triple-negative breast cancer [36]. We observed a positive correlation between the high-PPRS and sensitivity of chemotherapeutic drugs Cisplatin, Docetaxel, and Vinorelbine.

## 5. Conclusions

In summary, our study highlighted the importance of pyroptosis in LUAD and observed significantly different expression patterns between normal and LUAD samples. Importantly, we constructed a 7-gene prognostic signature related to pyroptosis, and the signature displayed a favorable performance in predicting LUAD prognosis. Notably, the differences on genomic features, enriched pathways and immune infiltration between PPRS-high and PPRS-low groups demonstrated a potential role of seven prognostic genes in the pyroptosis-related mechanism contributing to LUAD prognosis. The signature offered a comprehensive understanding of the correlation between immunotherapy/chemotherapy sensitivity of LUAD patients and cell pyroptosis. Our study provides a new insight for understanding pyroptosis-related mechanisms and the hope for developing new therapeutic drugs targeting pyroptosis for LUAD patients.

## Data Availability

The datasets used and/or analyzed during the current study are available in [GSE31210] at [https://www.ncbi.nlm.nih.gov/geo/query/acc.cgi?acc=GSE31210] and in [GSE72094] at [https://www.ncbi.nlm.nih.gov/geo/query/acc.cgi?acc=GSE72094].

## Supplementary Materials

Supplementary Materials Supplementary Materials. Figure S1. The workflow chart of this study. Figure S2. Correlation between Pyroptosis score and clinicopathological features and prognosis. (A) The correlation between pyroptosis score and different clinical features. (B, C) Univariate and multivariate Cox regression analysis showed the relationship between each clinicopathological feature and pyroptosis score and the prognosis of LUAD. (D) The OS of the low pyroptosis score group and the high pyroptosis score group based on the median pyroptosis score. (E) Pyroptosis scores of LUAD samples from TCGA were obtained by stratification according to age, gender, survival status, T stage, N SATGE, M stage and clinical stage. Figure S3. Functional analysis on 73 genes screened from the red module. The top 10 enriched terms were visualized. BP, biological process. CC, cellular component. MF, molecular function. Figure S4. TME of high-PPRS group and low-PPRS group in GSE31210 and GSE72094 cohorts. (A) The relative proportion of immune cells in the high-PPRS group and the low-PPRS group in the GSE31210 cohort. (B) Stromal score, immune score and ESTIMATE Score of high PPRS and low PPRS in GSE31210 cohort. (C) Differences in immune cell composition of different PPRS in GSE72094 cohort. (D) Stromal score, immune score and ESTIMATE score of high PPRS and low PPRS in GSE72094 cohort. Table S1. A list of 73 genes in the red module significantly associated with prognosis. Table S2. The correlation coefficients between KEGG pathways and PPRS.
