# Supplementary material for: Genomic and Immunological Characterization of Pyroptosis in Lung Adenocarcinoma
Source: J Oncol. 2022 Jul 27;2022:6905588. doi: 10.1155/2022/6905588 (PMC9348947; doi:10.1155/2022/6905588)
Supplement: Supplementary Materials — Figure S1. The workflow chart of this study. Figure S2. Correlation between Pyroptosis score and clinicopathological features and prognosis. (A) The correlation between pyroptosis score and different clinical features. (B, C) Univariate and multivariate Cox regression analysis showed the relationship between each clinicopathological feature and pyroptosis score and the prognosis of LUAD. (D) The OS of the low pyroptosis score group and the high pyroptosis score group based on the median pyroptosis score. (E) Pyroptosis scores of LUAD samples from TCGA were obtained by stratification according to age, gender, survival status, T stage, N SATGE, M stage and clinical stage. Figure S3. Functional analysis on 73 genes screened from the red module. The top 10 enriched terms were visualized. BP, biological process. CC, cellular component. MF, molecular function. Figure S4. TME of high-PPRS group and low-PPRS group in GSE31210 and GSE72094 cohorts. (A) The relative proportion of immune cells in the high-PPRS group and the low-PPRS group in the GSE31210 cohort. (B) Stromal score, immune score and ESTIMATE Score of high PPRS and low PPRS in GSE31210 cohort. (C) Differences in immune cell composition of different PPRS in GSE72094 cohort. (D) Stromal score, immune score and ESTIMATE score of high PPRS and low PPRS in GSE72094 cohort. Table S1. A list of 73 genes in the red module significantly associated with prognosis. Table S2. The correlation coefficients between KEGG pathways and PPRS. [file 6905588.f1.zip › 6905588.f1/Table S1.docx]

Table S1. A list of 73 genes in the red module significantly associated with prognosis

| Tag | p.value | HR | Low 95%CI | High 95%CI |
| --- | --- | --- | --- | --- |
| ABLIM3 | 0.027454 | 1.183629 | 1.018911 | 1.374975 |
| APOC2 | 0.038538 | 0.83949 | 0.711277 | 0.990815 |
| ATP6V1B2 | 0.044071 | 0.865934 | 0.752712 | 0.996186 |
| BTK | 0.003932 | 0.82236 | 0.719994 | 0.939279 |
| CCL13 | 0.045267 | 0.864634 | 0.749887 | 0.996939 |
| CCL17 | 0.03336 | 0.853428 | 0.737498 | 0.987582 |
| CCR2 | 0.000285 | 0.767286 | 0.664986 | 0.885323 |
| CD101 | 0.032908 | 0.853506 | 0.737903 | 0.987219 |
| CD180 | 0.049805 | 0.871098 | 0.758901 | 0.999882 |
| CD1B | 0.004784 | 0.798837 | 0.68343 | 0.933731 |
| CD1C | 0.001994 | 0.792209 | 0.683435 | 0.918295 |
| CD1E | 0.002946 | 0.79263 | 0.680049 | 0.92385 |
| CD300LF | 0.023584 | 0.849227 | 0.737182 | 0.978302 |
| CD33 | 0.04244 | 0.866323 | 0.754198 | 0.995117 |
| CD52 | 0.024016 | 0.850214 | 0.738461 | 0.978879 |
| CD74 | 0.026175 | 0.86228 | 0.756705 | 0.982585 |
| CLEC10A | 0.008104 | 0.823943 | 0.713904 | 0.950944 |
| CLEC4A | 0.049521 | 0.871488 | 0.75971 | 0.999712 |
| CLEC7A | 0.016507 | 0.848993 | 0.742648 | 0.970566 |
| CMTM7 | 0.006339 | 0.823179 | 0.715844 | 0.946608 |
| CPS1 | 0.000669 | 1.257699 | 1.102062 | 1.435317 |
| CX3CL1 | 0.048042 | 0.864458 | 0.748226 | 0.998746 |
| CXorf21 | 0.002738 | 0.810417 | 0.706285 | 0.929901 |
| CYP27A1 | 0.016073 | 0.8368 | 0.723804 | 0.967437 |
| DNAJC5B | 0.044826 | 0.857067 | 0.737185 | 0.996445 |
| EHD1 | 0.008563 | 1.233724 | 1.054906 | 1.442854 |
| FBP1 | 0.003165 | 0.809414 | 0.703373 | 0.931442 |
| FCER1A | 0.016109 | 0.833175 | 0.718089 | 0.966705 |
| FCRLB | 0.004548 | 1.213162 | 1.061575 | 1.386394 |
| FGD2 | 0.013823 | 0.832152 | 0.718907 | 0.963236 |
| FUCA1 | 0.007671 | 0.821156 | 0.710427 | 0.949144 |
| G0S2 | 0.013884 | 1.197119 | 1.037263 | 1.381609 |
| GIMAP1 | 0.025067 | 0.844433 | 0.728319 | 0.979059 |
| GMFG | 0.036841 | 0.865772 | 0.756193 | 0.991232 |
| GPR65 | 0.02336 | 0.850405 | 0.739258 | 0.978263 |
| HCLS1 | 0.038909 | 0.87165 | 0.765108 | 0.993028 |
| HLA-DMA | 0.001962 | 0.807419 | 0.705155 | 0.924514 |
| HLA-DMB | 0.007335 | 0.833652 | 0.729835 | 0.952236 |
| HLA-DPA1 | 0.033105 | 0.86526 | 0.757412 | 0.988463 |
| HLA-DPB1 | 0.014852 | 0.848581 | 0.743567 | 0.968425 |
| HLA-DQA1 | 0.026188 | 0.859443 | 0.752018 | 0.982212 |
| HLA-DQB2 | 0.026417 | 0.853301 | 0.741781 | 0.981588 |
| HLA-DRA | 0.018066 | 0.85265 | 0.747102 | 0.973109 |
| HLA-DRB1 | 0.02934 | 0.860672 | 0.752008 | 0.985037 |
| HLA-DRB5 | 0.011604 | 0.841409 | 0.735822 | 0.962148 |
| HPGDS | 0.002544 | 0.79526 | 0.685328 | 0.922825 |
| HRCT1 | 0.020204 | 1.185693 | 1.026947 | 1.368979 |
| HVCN1 | 0.034035 | 0.857004 | 0.743044 | 0.988441 |
| IGSF6 | 0.02943 | 0.857701 | 0.747028 | 0.984771 |
| INHA | 0.006859 | 1.204103 | 1.052416 | 1.377652 |
| KIAA1324 | 0.000917 | 0.778124 | 0.670856 | 0.902543 |
| KLF10 | 0.005895 | 1.229014 | 1.061234 | 1.42332 |
| LPXN | 0.043259 | 0.862886 | 0.747907 | 0.995542 |
| METRNL | 0.045 | 1.160518 | 1.003324 | 1.34234 |
| NLRC4 | 0.040449 | 0.860128 | 0.744687 | 0.993465 |
| P2RY12 | 0.009169 | 0.818441 | 0.703943 | 0.951563 |
| P2RY13 | 0.001722 | 0.794072 | 0.687449 | 0.917232 |
| PARVB | 0.011156 | 1.202775 | 1.042933 | 1.387114 |
| PLD4 | 0.011194 | 0.823024 | 0.708032 | 0.956691 |
| PPM1M | 0.00129 | 0.808659 | 0.710547 | 0.920318 |
| PRAM1 | 0.030406 | 0.850765 | 0.734954 | 0.984826 |
| RAB40B | 0.005766 | 0.810579 | 0.698309 | 0.9409 |
| RASGRP4 | 0.048473 | 0.864175 | 0.74753 | 0.999021 |
| RASSF4 | 0.043285 | 0.864215 | 0.750163 | 0.995606 |
| RPS6KA1 | 0.02553 | 0.846758 | 0.731745 | 0.979848 |
| RTN1 | 0.027459 | 0.848516 | 0.733239 | 0.981915 |
| SIRT4 | 0.038184 | 0.854984 | 0.737263 | 0.991502 |
| SLC14A2 | 0.044455 | 0.851012 | 0.727122 | 0.996009 |
| SPN | 0.028852 | 0.852669 | 0.739102 | 0.983687 |
| TLR7 | 0.011746 | 0.829477 | 0.717209 | 0.95932 |
| TMEM51 | 0.045449 | 1.161943 | 1.003044 | 1.346015 |
| TNIP1 | 0.030124 | 1.177439 | 1.015829 | 1.36476 |
| UNC13D | 0.044045 | 1.166078 | 1.004106 | 1.354179 |
