# Supplementary material for: Genomic and Immunological Characterization of Pyroptosis in Lung Adenocarcinoma
Source: J Oncol. 2022 Jul 27;2022:6905588. doi: 10.1155/2022/6905588 (PMC9348947; doi:10.1155/2022/6905588)
Supplement: Supplementary Materials — Figure S1. The workflow chart of this study. Figure S2. Correlation between Pyroptosis score and clinicopathological features and prognosis. (A) The correlation between pyroptosis score and different clinical features. (B, C) Univariate and multivariate Cox regression analysis showed the relationship between each clinicopathological feature and pyroptosis score and the prognosis of LUAD. (D) The OS of the low pyroptosis score group and the high pyroptosis score group based on the median pyroptosis score. (E) Pyroptosis scores of LUAD samples from TCGA were obtained by stratification according to age, gender, survival status, T stage, N SATGE, M stage and clinical stage. Figure S3. Functional analysis on 73 genes screened from the red module. The top 10 enriched terms were visualized. BP, biological process. CC, cellular component. MF, molecular function. Figure S4. TME of high-PPRS group and low-PPRS group in GSE31210 and GSE72094 cohorts. (A) The relative proportion of immune cells in the high-PPRS group and the low-PPRS group in the GSE31210 cohort. (B) Stromal score, immune score and ESTIMATE Score of high PPRS and low PPRS in GSE31210 cohort. (C) Differences in immune cell composition of different PPRS in GSE72094 cohort. (D) Stromal score, immune score and ESTIMATE score of high PPRS and low PPRS in GSE72094 cohort. Table S1. A list of 73 genes in the red module significantly associated with prognosis. Table S2. The correlation coefficients between KEGG pathways and PPRS. [file 6905588.f1.zip › 6905588.f1/Table S2.pdf]

Table S2. The correlation coefficients between KEGG pathways and PPRS.

V1	correlation coefficients
KEGG_N_GLYCAN_BIOSYNTHESIS	0.156570618
KEGG_OTHER_GLYCAN_DEGRADATION	-0.20593355
KEGG_O_GLYCAN_BIOSYNTHESIS	-0.0529296
KEGG_GLYCOSAMINOGLYCAN_DEGRADATION	-0.04049898
KEGG_GLYCOSAMINOGLYCAN_BIOSYNTHESIS_KERATAN_SULFATE	0.077555828
KEGG_GLYCEROLIPID_METABOLISM	-0.0489975
KEGG_GLYCOSYLPHOSPHATIDYLINOSITOL_GPI_ANCHOR_BIOSYNTHESIS	0.119905081
KEGG_GLYCEROPHOSPHOLIPID_METABOLISM	-0.2173353
KEGG_ETHER_LIPID_METABOLISM	-0.32981163
KEGG_ARACHIDONIC_ACID_METABOLISM	-0.29123188
KEGG_LINOLEIC_ACID_METABOLISM	-0.23667443
KEGG_ALPHA_LINOLENIC_ACID_METABOLISM	-0.3425803
KEGG_SPHINGOLIPID_METABOLISM	-0.05928553
KEGG_GLYCOSPHINGOLIPID_BIOSYNTHESIS_LACTO_AND_NEOLACTO_SERIES	0.233780803
KEGG_GLYCOSPHINGOLIPID_BIOSYNTHESIS_GLOBO_SERIES	0.103893464
KEGG_GLYCOSPHINGOLIPID_BIOSYNTHESIS_GANGLIO_SERIES	-0.25813095
KEGG_RIBOFLAVIN_METABOLISM	0.128452051
KEGG_NICOTINATE_AND_NICOTINAMIDE_METABOLISM	0.050657337

KEGG_PANTOTHENATE_AND_COA_BIOSYNTHESIS	-0.05319159
KEGG_AMINOACYL_TRNA_BIOSYNTHESIS	0.341363221
KEGG_BASAL_TRANSCRIPTION_FACTORS	0.36050053
KEGG_NON_HOMOLOGOUS_END_JOINING	0.276655419
KEGG_SNARE_INTERACTIONS_IN_VESICULAR_TRANSPORT	-0.05256503
KEGG_LYSOSOME	-0.2243905
KEGG_CARDIAC_MUSCLE_CONTRACTION	-0.10081904
KEGG_RENIN_ANGIOTENSIN_SYSTEM	-0.22120435
KEGG_CIRCADIAN_RHYTHM_MAMMAL	-0.13892825
KEGG_TASTE_TRANSDUCTION	-0.3011986
KEGG_PROXIMAL_TUBULE_BICARBONATE_RECLAMATION	-0.17337574
KEGG_PATHOGENIC_ESCHERICHIA_COLI_INFECTION	-0.08439265
KEGG_SYSTEMIC_LUPUS_ERYTHEMATOSUS	-0.10030518
KEGG_PRIMARY_IMMUNODEFICIENCY	-0.27039679
KEGG_HYPERTROPHIC_CARDIOMYOPATHY_HCM	-0.28707205
KEGG_ARRHYTHMOGENIC_RIGHT_VENTRICULAR_CARDIOMYOPATHY_ARVC	-0.20470237
KEGG_DILATED_CARDIOMYOPATHY	-0.30808974
KEGG_GLYCOLYSIS_GLUCONEOGENESIS	-0.00299956
KEGG_CITRATE_CYCLE_TCA_CYCLE	0.184218638
KEGG_PENTOSE_PHOSPHATE_PATHWAY	0.280946461

KEGG_PENTOSE_AND_GLUCURONATE_INTERCONVERSIONS	0.024142277
KEGG_FRUCTOSE_AND_MANNOSE_METABOLISM	0.273279864
KEGG_GALACTOSE_METABOLISM	0.231167104
KEGG_ASCORBATE_AND_ALDARATE_METABOLISM	-0.01646785
KEGG_FATTY_ACID_METABOLISM	-0.24326616
KEGG_STEROID_BIOSYNTHESIS	0.156478558
KEGG_PRIMARY_BILE_ACID_BIOSYNTHESIS	-0.2919175
KEGG_STEROID_HORMONE_BIOSYNTHESIS	-0.01936393
KEGG_OXIDATIVE_PHOSPHORYLATION	0.19950154
KEGG_PURINE_METABOLISM	0.324206174
KEGG_PYRIMIDINE_METABOLISM	0.489313517
KEGG_ALANINE_ASPARTATE_AND_GLUTAMATE_METABOLISM	0.185680845
KEGG_GLYCINE_SERINE_AND_THREONINE_METABOLISM	0.020628971
KEGG_CYSTEINE_AND_METHIONINE_METABOLISM	0.201199896
KEGG_VALINE_LEUCINE_AND_ISOLEUCINE_DEGRADATION	-0.1592765
KEGG_VALINE_LEUCINE_AND_ISOLEUCINE_BIOSYNTHESIS	0.14456553
KEGG_LYSINE_DEGRADATION	0.239595031
KEGG_ARGININE_AND_PROLINE_METABOLISM	0.014792918
KEGG_HISTIDINE_METABOLISM	-0.09364683
KEGG_TYROSINE_METABOLISM	-0.09342222

KEGG_PHENYLALANINE_METABOLISM	-0.04898521
KEGG_TRYPTOPHAN_METABOLISM	-0.21088052
KEGG_BETA_ALANINE_METABOLISM	-0.25821977
KEGG_SELENOAMINO_ACID_METABOLISM	-0.1414204
KEGG_GLUTATHIONE_METABOLISM	0.068342307
KEGG_STARCH_AND_SUCROSE_METABOLISM	0.148245777
KEGG_AMINO_SUGAR_AND_NUCLEOTIDE_SUGAR_METABOLISM	0.119357334
KEGG_GLYCOSAMINOGLYCAN_BIOSYNTHESIS_CHONDROITIN_SULFATE	0.033069254
KEGG_GLYCOSAMINOGLYCAN_BIOSYNTHESIS_HEPARAN_SULFATE	0.030040627
KEGG_INOSITOL_PHOSPHATE_METABOLISM	-0.22277209
KEGG_PYRUVATE_METABOLISM	-0.02862573
KEGG_GLYOXYLATE_AND_DICARBOXYLATE_METABOLISM	0.379193672
KEGG_PROPANOATE_METABOLISM	-0.16433236
KEGG_BUTANOATE_METABOLISM	0.019235311
KEGG_ONE_CARBON_POOL_BY_FOLATE	0.384541994
KEGG_FOLATE_BIOSYNTHESIS	0.207393336
KEGG_RETINOL_METABOLISM	-0.06608114
KEGG_PORPHYRIN_AND_CHLOROPHYLL_METABOLISM	0.153301378
KEGG_TERPENOID_BACKBONE_BIOSYNTHESIS	0.221372728
KEGG_LIMONENE_AND_PINENE_DEGRADATION	-0.07727639

KEGG_NITROGEN_METABOLISM	0.046322307
KEGG_SULFUR_METABOLISM	-0.02693503
KEGG_METABOLISM_OF_XENOBIOTICS_BY_CYTOCHROME_P450	-0.04787658
KEGG_DRUG_METABOLISM_CYTOCHROME_P450	-0.21353056
KEGG_DRUG_METABOLISM_OTHER_ENZYMES	0.240035161
KEGG_BIOSYNTHESIS_OF_UNSATURATED_FATTY_ACIDS	0.2683466
KEGG_ABC_TRANSPORTERS	-0.29889391
KEGG_RIBOSOME	0.145728332
KEGG_RNA_DEGRADATION	0.269090114
KEGG_RNA_POLYMERASE	0.326870243
KEGG_DNA_REPLICATION	0.446608546
KEGG_SPLICEOSOME	0.372356293
KEGG_PROTEASOME	0.348254559
KEGG_PROTEIN_EXPORT	0.075423374
KEGG_PPAR_SIGNALING_PATHWAY	-0.22072707
KEGG_BASE_EXCISION_REPAIR	0.429131899
KEGG_NUCLEOTIDE_EXCISION_REPAIR	0.433693928
KEGG_MISMATCH_REPAIR	0.406908214
KEGG_HOMOLOGOUS_RECOMBINATION	0.445059936
KEGG_MAPK_SIGNALING_PATHWAY	-0.26375267

KEGG_ERBB_SIGNALING_PATHWAY	-0.09108111
KEGG_CALCIUM_SIGNALING_PATHWAY	-0.29414161
KEGG_CYTOKINE_CYTOKINE_RECEPTOR_INTERACTION	-0.23355093
KEGG_CHEMOKINE_SIGNALING_PATHWAY	-0.26641299
KEGG_PHOSPHATIDYLINOSITOL_SIGNALING_SYSTEM	-0.29001522
KEGG_NEUROACTIVE_LIGAND_RECEPTOR_INTERACTION	-0.19705701
KEGG_CELL_CYCLE	0.448053471
KEGG_OOCYTE_MEIOSIS	0.341511301
KEGG_P53_SIGNALING_PATHWAY	0.353900469
KEGG_UBIQUITIN_MEDIATED_PROTEOLYSIS	0.241368176
KEGG_REGULATION_OF_AUTOPHAGY	0.009279303
KEGG_ENDOCYTOSIS	-0.1793037
KEGG_PEROXISOME	-0.0786546
KEGG_MTOR_SIGNALING_PATHWAY	-0.18188439
KEGG_APOPTOSIS	-0.15725897
KEGG_VASCULAR_SMOOTH_MUSCLE_CONTRACTION	-0.433634
KEGG_WNT_SIGNALING_PATHWAY	-0.12686868
KEGG_DORSO_VENTRAL_AXIS_FORMATION	-0.1514633
KEGG_NOTCH_SIGNALING_PATHWAY	0.00442705
KEGG_HEDGEHOG_SIGNALING_PATHWAY	-0.12161257

KEGG_TGF_BETA_SIGNALING_PATHWAY	-0.15730945
KEGG_AXON_GUIDANCE	-0.06009692
KEGG_VEGF_SIGNALING_PATHWAY	-0.29924654
KEGG_FOCAL_ADHESION	-0.13769362
KEGG_ECM_RECEPTOR_INTERACTION	-0.08878167
KEGG_CELL_ADHESION_MOLECULES_CAMS	-0.36150436
KEGG_ADHERENS_JUNCTION	-0.1085039
KEGG_TIGHT_JUNCTION	-0.23618667
KEGG_GAP_JUNCTION	-0.11617756
KEGG_COMPLEMENT_AND_COAGULATION_CASCADES	-0.25686714
KEGG_ANTIGEN_PROCESSING_AND_PRESENTATION	-0.18405999
KEGG_TOLL_LIKE_RECEPTOR_SIGNALING_PATHWAY	-0.13459583
KEGG_NOD_LIKE_RECEPTOR_SIGNALING_PATHWAY	-0.09569688
KEGG_RIG_I_LIKE_RECEPTOR_SIGNALING_PATHWAY	-0.03146496
KEGG_CYTOSOLIC_DNA_SENSING_PATHWAY	-0.00304626
KEGG_JAK_STAT_SIGNALING_PATHWAY	-0.3104889
KEGG_HEMATOPOIETIC_CELL_LINEAGE	-0.32813924
KEGG_NATURAL_KILLER_CELL_MEDIATED_CYTOTOXICITY	-0.21963467
KEGG_T_CELL_RECEPTOR_SIGNALING_PATHWAY	-0.2839812
KEGG_B_CELL_RECEPTOR_SIGNALING_PATHWAY	-0.28549862

KEGG_FC_EPSILON_RI_SIGNALING_PATHWAY	-0.4719179
KEGG_FC_GAMMA_R_MEDIATED_PHAGOCYTOSIS	-0.21922834
KEGG_LEUKOCYTE_TRANSENDOTHELIAL_MIGRATION	-0.35396271
KEGG_INTESTINAL_IMMUNE_NETWORK_FOR_IGA_PRODUCTION	-0.31817549
KEGG_LONG_TERM_POTENTIATION	-0.30024412
KEGG_NEUROTROPHIN_SIGNALING_PATHWAY	-0.2162117
KEGG_LONG_TERM_DEPRESSION	-0.40730663
KEGG_OLFACTORY_TRANSDUCTION	-0.09590757
KEGG_REGULATION_OF_ACTIN_CYTOSKELETON	-0.15777587
KEGG_INSULIN_SIGNALING_PATHWAY	-0.10765159
KEGG_GNRH_SIGNALING_PATHWAY	-0.36885851
KEGG_PROGESTERONE_MEDIATED_OOCYTE_MATURATION	0.288882133
KEGG_MELANOGENESIS	-0.20056077
KEGG_ADIPOCYTOKINE_SIGNALING_PATHWAY	-0.1007399
KEGG_TYPE_II_DIABETES_MELLITUS	-0.27890847
KEGG_TYPE_I_DIABETES_MELLITUS	-0.21584549
KEGG_MATURITY_ONSET_DIABETES_OF_THE_YOUNG	-0.00037029
KEGG_ALDOSTERONE_REGULATED_SODIUM_REABSORPTION	-0.34970401
KEGG_VASOPRESSIN_REGULATED_WATER_REABSORPTION	-0.31902898
KEGG_ALZHEIMERS_DISEASE	0.187227374

KEGG_PARKINSONS_DISEASE	0.209800467
KEGG_AMYOTROPHIC_LATERAL_SCLEROSIS_ALS	0.176529055
KEGG_HUNTINGTONS_DISEASE	0.182654035
KEGG_PRION_DISEASES	-0.20475932
KEGG_VIBRIO_CHOLERAE_INFECTION	-0.19454213
KEGG_EPITHELIAL_CELL_SIGNALING_IN_HELICOBACTER_PYLORI_INFECTION	-0.05931305
KEGG_LEISHMANIA_INFECTION	-0.23710263
KEGG_PATHWAYS_IN_CANCER	-0.04861995
KEGG_COLORECTAL_CANCER	-0.11247282
KEGG_RENAL_CELL_CARCINOMA	-0.04431335
KEGG_PANCREATIC_CANCER	0.023214688
KEGG_ENDOMETRIAL_CANCER	-0.23410871
KEGG_GLIOMA	-0.09182289
KEGG_PROSTATE_CANCER	-0.12583428
KEGG_THYROID_CANCER	-0.00203148
KEGG_BASAL_CELL_CARCINOMA	-0.11765695
KEGG_MELANOMA	-0.04160705
KEGG_BLADDER_CANCER	0.184524704
KEGG_CHRONIC_MYELOID_LEUKEMIA	-0.01464231
KEGG_ACUTE_MYELOID_LEUKEMIA	-0.28626294

KEGG_SMALL_CELL_LUNG_CANCER	0.064303043
KEGG_NON_SMALL_CELL_LUNG_CANCER	-0.13697794
KEGG_ASTHMA	-0.33460206
KEGG_AUTOIMMUNE_THYROID_DISEASE	-0.26102778
KEGG_ALLOGRAFT_REJECTION	-0.24636694
KEGG_GRAFT_VERSUS_HOST_DISEASE	-0.18629625
KEGG_VIRAL_MYOCARDITIS	-0.33568
